# Non-medicinal oral contrast in upper abdominal MRI for MR-guided radiotherapy: A scoping review

**DOI:** 10.1016/j.radi.2025.01.003

**Published:** 2025-03

**Authors:** M.R. Beasley, A.M. Henry, J. Bestall, V.P. Cosgrove, L.J. Murray, C. Burnett

**Affiliations:** a Radiotherapy, Leeds Cancer Centre, Leeds Teaching Hospitals NHS Trust, UK; b Leeds Institute of Medical Research, University of Leeds, Leeds, UK; c Clinical Oncology, Leeds Cancer Centre, Leeds Teaching Hospitals NHS Trust, UK; d Leeds Institute of Health Sciences, University of Leeds, Leeds, UK; e Medical Physics and Engineering, Leeds Cancer Centre, Leeds Teaching Hospitals NHS Trust, Leeds, UK; f NIHR Leeds Biomedical Research Centre, Leeds, UK

**Keywords:** Non-medicinal oral contrast, MRI, Luminal contrast, Radiotherapy

## Abstract

**Introduction:**

Using non-medicinal oral contrast agents may aid safe delivery of magnetic resonance image-guided (MR-guided) radiotherapy by improving the ability to visualise and avoid excessive radiation dose to adjacent bowel/stomach. This scoping review aims to map the literature on non-medicinal oral contrasts used in upper-abdominal diagnostic or therapeutic magnetic resonance imaging (MRI) to find potential candidates for employing in MR-guided radiotherapy and identify gaps in knowledge for further study.

**Methods:**

A scoping review of non-medicinal oral contrast used in upper-abdominal MRI research followed a pre-defined protocol based on Arksey and O’Malley’s framework. Data were charted and reported in accordance with the Preferred Reporting Items for Systematic reviews and Meta-Analyses for Scoping Reviews reporting guidelines.

**Results:**

Forty-seven studies from 1955 screened abstracts were charted. Thirty-one distinct non-medicinal oral contrast were identified, used primarily to enhance tissue visualisation (89 %) or observe motility (11 %) in diagnostic studies. All studies reported to be predominantly quantitative; only 13 % included participant experience via questionnaires and none used qualitative methods. No studies have examined the efficacy of non-medicinal oral contrasts in MR-guided radiotherapy planning or delivery.

**Conclusion:**

Non-medicinal oral contrasts have been extensively investigated in diagnostic MRI to enhance gastrointestinal visualisation and assess motility. However, non-medicinal oral contrasts have not been investigated in the context of radiotherapy planning and treatment. Qualitative evaluation of the patient experience of non-medicinal oral contrasts in magnetic resonance image-guided radiotherapy should be considered alongside studies quantifying the potential clinical benefit.

**Implications for practice:**

This review summarises the properties of non-medicinal oral contrasts and identifies critical gaps in the current evidence, particularly the absence of qualitative research in this domain and the unexplored potential for their application in MR-guided radiotherapy planning and delivery.

## Introduction

Magnetic resonance imaging (MRI) is recognised for its superior soft-tissue discrimination compared to computed tomography (CT), making it instrumental in cancer diagnosis and treatment planning.^1^^,^^2^ The relatively recent integration of MRI into radiotherapy pathways, particularly with the advent of magnetic resonance (MR)-simulators and MR-linacs, has the capability of enhancing tumour and normal tissue visualisation to aid clinical/radiation oncologists in structure definition and radiotherapy treatment planning.^3^, ^4^, ^5^, ^6^, ^7^, ^8^ Additionally, functional MRI techniques offer the potential for personalised dose distributions and adaptive radiotherapy through imaging biological information, with the potential to improve patient outcomes.^9^^,^^10^

Utilising MRI, especially in MR-guided upper abdominal radiotherapy, presents challenges such as interobserver variation in accurately delineating tumours and organs-at-risk (OAR).^4^ These difficulties arise from the complexity of abdominal anatomy, tissue conspicuity, and the impact of motion on image quality.^4^ Advances in diagnostic strategies have enhanced the visualisation of the gastrointestinal (GI) tract, often through the oral administration of contrast agents that modify luminal signal characteristics, facilitating the identification of specific anatomical structures and/or pathologies.^11^^,^^12^ Depending on the type of oral contrast agent used and the specific MRI sequence employed, the resultant luminal signal on MRI may appear either hyperintense or hypointense relative to reference tissues such as the psoas muscle. These agents can range from pharmaceutical medicines to naturally derived substances and foods such as fruit juices and milk-based products. The literature suggests that ideal oral contrast agents should not only improve visualisation of the targeted anatomy, but also be palatable and cost-effective.^6^^,^^11^^,^^13^, ^14^, ^15^

When selecting oral contrasts to optimise image quality, it is also important to consider their impact on patient experience. While certain medicinal MRI contrast agents are often effective, they can cause side effects, such as GI discomfort.^16^ To mitigate these effects, non-medicinal oral contrasts (NMOCs) might offer a promising alternative, potentially reducing adverse reactions whilst improving patient comfort and adherence.^6^^,^^15^^,^^17^^,^^18^ The application of oral contrast agents, whether medicinal or not, in upper-abdominal MR-guided radiotherapy appears to have not been investigated.

### Objectives

This scoping review aims to systematically map current knowledge by identifying the types and characteristics of NMOCs that have been studied. The review has been designed to reveal gaps in existing knowledge in relation to use in radiotherapy and highlight variations in effectiveness, patient experience and acceptability of NMOCs in this context.

## Methods

A pre-defined protocol based on Arksey and O'Malley’s framework was followed and published on the Open Science Framework.^19^^,^^20^ The Preferred Reporting Items for Systematic reviews and Meta-Analyses Protocols (PRISMA-P) statement and the extension for Scoping Reviews (PRISMA-ScR) reporting guidelines was followed in producing the written report.^21^ Since this study was extracting data from existing publicly available published papers, ethical approval was unnecessary.

### Search and screening strategy

A search strategy (Supplementary material A) was developed with assistance from an information specialist. In line with the recommendations of the Joanna Briggs Institute, with respect to the broad topic and review question, the objectives were assigned to the population, concept and context (PCC) framework shown in Table 1.^22^ Manual bidirectional citation searching was undertaken to ensure a thorough search and identify studies potentially missed by bibliographic database search methods.^23^ The literature was searched from inception to 01/05/2024 using MEDLINE (Ovid), EMBASE, CINAHL (EBSCOhost), CAB Abstracts databases. To aid with mitigating publication bias, additional sources included Google Scholar and ProQuest Dissertations & Theses. The search included potentially relevant research in all languages. Where the full text was unavailable or not in English, it was excluded at the full text review stage. Animal studies were excluded. All database records were transferred to EndNote 20 (Clarivate, Philadelphia, PA) and duplicates were removed using the Bramer method.^24^^,^^25^

**Table 1:** Objective PCC criteria.

Objective	Inclusion criteria	Exclusion criteria
Population	Adults aged 18 or over, any condition, any country.	Animal studies.
Concept	Upper-abdominal MRI with natural (non-medicinal) oral contrast agent ingestion – how were they perceived and when, how, and why were they used?	Non-medicinal oral contrast not used as part of the empirical evaluation studied.
Context	Studies to have taken place in a hospital - clinical or academic.	Full text unavailable. Studies not published in English.

Studies published in peer reviewed journals which met the eligibility criteria in Table 1 were included for title and abstract screening. Screening was conducted by the first author, with a minimum proportion of 10 % of results being independently checked by a second reviewer (CB) using Covidence systematic review software (Veritas Health Innovation, Melbourne, Australia).^26^ Testing for inter-screening reliability of the screened proportion was quantified using Cohen’s kappa scores.^27^ Disagreements were countered with consensus. Reasons for full-text exclusion were recorded.

### Data charting

An *a priori* charting framework form was developed for intended use on the first 10 % of the included studies and was modified until the codes were coherent and explicit. Cohen’s Kappa statistic was used to test the level agreement. The objectives of this scoping review did not encompass the assignment of quality grading scores. Utilising risk of bias tools was considered unnecessary and potentially inappropriate due to the substantial methodological variability across the included studies.^28^ Data was charted and aggregated to provide an account of the utilisation of NMOC use in upper-abdominal MRI. Data items extracted included: oral contrasts tested; type of publication, year of publication, country, method (*in vitro*, *in vivo*); participants; themes; aims; design; funding; conflicts of interest; inclusion and exclusion criteria; participant preparation; intervention details; MRI acquisition details (including manufacturer and sequences used); participant positioning; if IV contrast was used; anatomical site of interest; reference anatomy; plane of imaging; signal properties and appearance; quantitative imaging results; methods of evaluation; statistical analysis details; reported outcomes; participant experience details and outcome. Studies were grouped by the studies by the NMOCs analysed, and summarised the thematic type of aims (e.g. visualisation or motility), designs, and populations, along with metrics used and findings.

## Results

### Literature search results

**Figure 1:**
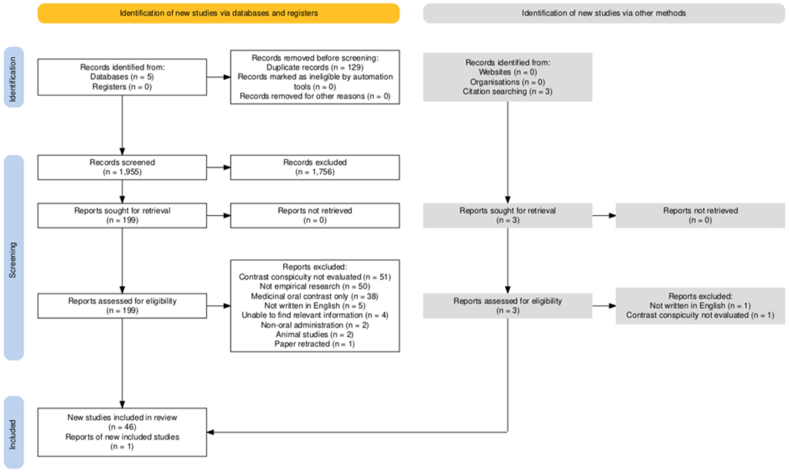
PRISMA flow diagram of scoping review screening process.

Fig. 1 presents the PRISMA flow diagram illustrating the search and screening process. Initially, 1955 unique citations were identified. Following the review of titles and abstracts, 1756 citations were excluded. The first author screened 77 % of abstracts, with 449 out of 1955 (23 %) being subjected to independent screening by two reviewers, resulting in a proportionate agreement of 97.3 % and a Cohen’s Kappa coefficient of 0.88. Full-text screening was performed on 199 articles, of which 153 were excluded for failing to meet the inclusion criteria, and one article was retracted due to plagiarism. An additional article was incorporated into the full-text review following citation searching. A total of six studies were excluded due to not being written or translated into English.^29^, ^30^, ^31^, ^32^, ^33^, ^34^ A final total of 47 studies were included for data charting, comprising of 44 research articles, two conference abstracts, and one short communication paper. Characteristics of the evidence including details of funding sources and statements of conflicts of interest are shown in Supplementary material B.

### Frequency of non-medicinal oral contrasts (NMOCs) reported in the literature

**Figure 2:**
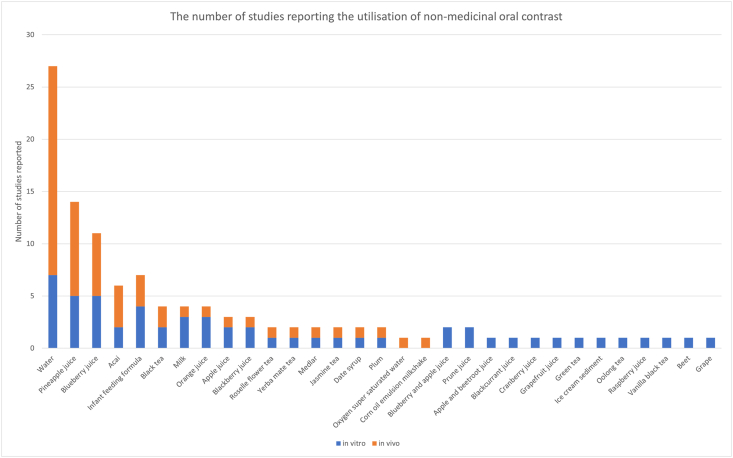
The number of studies reporting the utilisation of NMOCs. Each bar represents a specific contrast medium, with blue segments indicating the number of NMOCs studied *in vitro* studies and orange segments representing the number of NMOCs studied *in vivo*. (For interpretation of the references to color in this figure legend, the reader is referred to the Web version of this article.)

A total of 31 distinct NMOCs were utilised across the studies reviewed. The frequency of studies reporting the use of various NMOCs, distinguishing between *in vitro* (NMOCs imaged in phantoms) and *in vivo* applications (NMOCs imaged in humans) is summarised in Fig. 2.

All included studies performed *in vivo* testing in humans; however, 18 of these studies (58 % of NMOCs used) reported preliminary *in vitro* evaluations. *In vitro* evaluations, although not the focus of our review, were reported to provide the foundational data that informed the progression to *in vivo* applications. Twenty-nine studies (62 %) reported the use of oral contrasts *in vivo* only and publications often included more than one candidate of NMOC for evaluation.

Water was the most frequently studied NMOC, with a total of 27 studies. Fruit juices included pineapple juice (10 studies) and blueberry juice (8 studies) with a relatively balanced distribution between *in vitro* and *in vivo* study components. Other fruit juices such as acai juice appeared in multiple studies but with far fewer total reports compared to pineapple and blueberry juices. Infant feeding formula featured in earlier MRI contrast studies (three were *in vivo* studies) between the years 1989 and 1992. Less commonly studied media, including various teas (e.g. green tea, jasmine tea, yerba mate tea), and other substances such as ice cream emulsion or oxygen-supersaturated water) have only been reported once each in the literature. The specific NMOCs used, grouped by food types along with their corresponding references are detailed in Table 2.

**Table 2:** NMOC types studied. The NMOCs have been grouped by food type and their reference and publication count by subtype.

NMOC	Visualisation Publications	Motility Publications	Total publications
Water			28
Water	25^13^^,^^16^^,^^35^, ^36^, ^37^, ^38^, ^39^, ^40^, ^41^, ^42^, ^43^, ^44^, ^45^, ^46^, ^47^, ^48^, ^49^, ^50^, ^51^, ^52^, ^53^, ^54^, ^55^, ^56^, ^57^, ^58^	3^44^^,^^59^^,^^60^	
Oxygen super-saturated water	1^51^		
Fruit/vegetable juice			21
Pineapple juice	9^13^^,^^17^^,^^35^^,^^36^^,^^41^^,^^42^^,^^61^, ^62^, ^63^	1^64^	
Blueberry juice	8^13^^,^^17^^,^^36^^,^^42^^,^^48^^,^^65^, ^66^, ^67^		
Acai juice	5^38^^,^^39^^,^^68^, ^69^, ^70^		
Orange juice	3^13^^,^^35^^,^^36^		
Apple juice	2^13^^,^^42^	1^71^	
Blackberry juice	2^35^^,^^42^		
Blackcurrant juice	1^35^		
Blueberry and apple juice	2^13^^,^^35^		
Prune juice	2^13^^,^^35^		
Apple and beetroot juice	1^35^		
Beet juice	1^42^		
Cranberry juice	1^13^		
Date syrup	1^72^		
Grape juice	1^42^		
Grapefruit juice	1^13^		
Medlar	1^39^		
Plum	1^42^		
Raspberry juice	1^35^		
Milk/emulsion			10
Milk	5^13^^,^^37^^,^^41^^,^^72^^,^^73^		
Ice cream sediment	1^37^		
12 % corn oil emulsion	1^74^		
Infant feeding formula	4^35^^,^^37^^,^^43^^,^^73^		
Teas			5
Black tea	3^55^^,^^75^^,^^76^		
Green tea	1^55^		
Jasmine tea	1^55^		
Oolong tea	1^55^		
Roselle flower tea	1^77^		
Vanilla black tea	1^55^		
Yerba mate	1^78^		

### Study aims

All studies could be broadly categorised into two themes based on their aims: investigating either motility (5/47 (11 %)^44^^,^^59^^,^^60^^,^^64^^,^^71^ or tissue visualisation (42/47, 89 %).^13^^,^^16^^,^^17^^,^^35^, ^36^, ^37^, ^38^, ^39^, ^40^, ^41^, ^42^, ^43^^,^^45^, ^46^, ^47^, ^48^, ^49^, ^50^, ^51^, ^52^, ^53^, ^54^, ^55^, ^56^, ^57^, ^58^^,^^61^, ^62^, ^63^^,^^65^, ^66^, ^67^, ^68^, ^69^, ^70^^,^^72^, ^73^, ^74^, ^75^, ^76^, ^77^, ^78^ Twenty-eight percent of the tissue visualisation studies (13/47) specifically aimed to investigate luminal signal suppression properties of NMOC for T2-weighted magnetic resonance cholangiopancreatography (MRCP) sequences.^13^^,^^17^^,^^55^^,^^61^^,^^62^^,^^67^, ^68^, ^69^, ^70^^,^^72^^,^^75^, ^76^, ^77^

### Study designs

The most common study design was an uncontrolled before-after study, where outcomes were measured before and after the intervention in the same group of participants, without the use of a control or comparison group (35/47, 74 %).^13^^,^^16^^,^^17^^,^^35^^,^^38^, ^39^, ^40^, ^41^, ^42^^,^^44^, ^45^, ^46^, ^47^, ^48^^,^^51^, ^52^, ^53^, ^54^, ^55^^,^^57^, ^58^, ^59^, ^60^, ^61^^,^^65^, ^66^, ^67^, ^68^, ^69^, ^70^^,^^72^^,^^74^, ^75^, ^76^, ^77^ One study described itself as an ‘observational comparative analytical study,’ however it was also recorded as an uncontrolled before-after study, as it evaluated before and after images following a pineapple juice intervention.^61^ There were five uncontrolled studies (11 %) without comparators,^49^^,^^50^^,^^64^^,^^73^^,^^78^ three controlled before-after studies (7 %),^36^^,^^56^^,^^62^ one retrospective uncontrolled study (2 %),^43^ one study that included both an uncontrolled before-after design and an uncontrolled study (2 %),^37^ and one randomised controlled trial (2 %).^71^ One study was recorded as a comparative cohort study despite the authors stating it was a case–control study, since it tracked outcomes of group exposed to different treatments forward in time, rather than looking backward and there being no control group.^63^ Supplementary material C shows the breakdown of studies by design and their participant types.

### Study populations

Twenty-six out of forty-seven (55 %) studies conducted their research in healthy volunteers,^13^^,^^16^^,^^17^^,^^39^, ^40^, ^41^^,^^44^^,^^47^, ^48^, ^49^, ^50^, ^51^^,^^54^^,^^55^^,^^57^^,^^59^^,^^60^^,^^64^, ^65^, ^66^^,^^68^^,^^71^^,^^74^, ^75^, ^76^, ^77^ while 6/47 (13 %) combining healthy volunteers for preliminary research before progressing to utilising patient populations^36^^,^^43^^,^^66^^,^^70^^,^^77^^,^^78^ and 15/47 (30 %) conducting research exclusively in patient populations.^42^^,^^45^^,^^46^^,^^52^^,^^53^^,^^56^^,^^58^^,^^61^, ^62^, ^63^^,^^67^^,^^69^^,^^72^^,^^73^^,^^77^ Nine studies (35 %) reported the mean age of their healthy volunteer participants,^13^^,^^16^^,^^36^^,^^40^^,^^44^^,^^50^^,^^57^^,^^65^^,^^74^ and 12 studies reported the mean age of their patient participants.^45^^,^^46^^,^^53^^,^^58^^,^^61^^,^^67^^,^^69^^,^^70^^,^^72^^,^^73^^,^^75^^,^^77^

### Non-medicinal oral contrast signal properties

**Figure 3:**
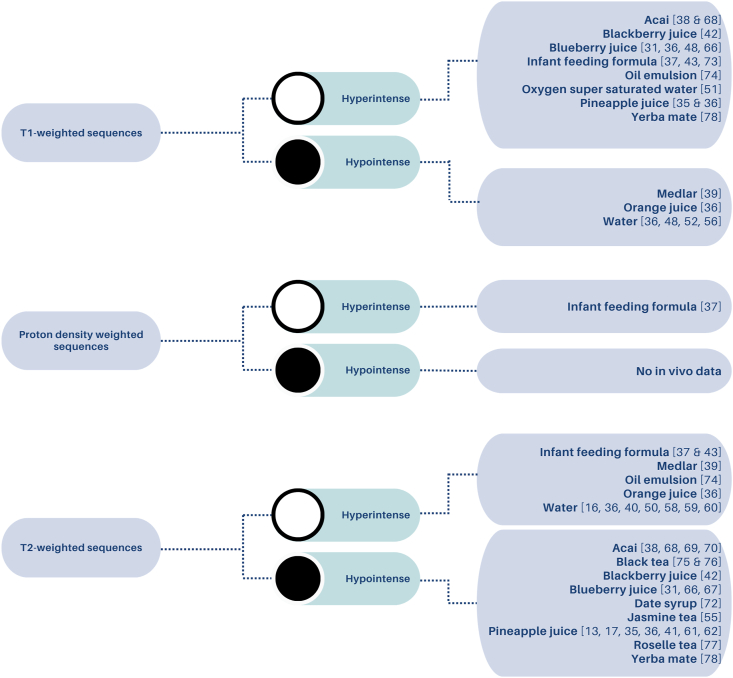
Tree diagram of the reported NMOC signal properties when using T1w, Proton Density w, and T2w sequences *in vivo*.

Fig. 3 presents an overview of the signal appearance properties of the NMOCs when imaged using different MRI weighting sequences reported in 37/47 (78.7 %) studies.

### T1-weighted sequences

In T1-weighted MRI sequences, agents such as acai, blueberry juice, and pineapple juice reportedly exhibited hyperintense signals, indicating a shorter T1.^36^^,^^48^^,^^65^^,^^66^ Conversely, medlar, orange juice, and water demonstrated hypointense signals, reflecting a longer T1.^36^^,^^39^^,^^48^^,^^52^^,^^56^^,^^58^^,^^74^

### Proton density-weighted sequences

Infant feeding formula was the only agent reported with a proton density weighted sequence, and it was noted to be hyperintense.^37^

### T2-weighted sequences

In T2-weighted sequences, agents like water and infant feeding formula appeared hyperintense.^16^^,^^36^^,^^40^^,^^50^^,^^58^, ^59^, ^60^ Conversely, teas such as black tea and yerba mate were reported as hypointense.^68^, ^69^, ^70^^,^^75^^,^^76^

Nine out of forty-seven (19 %) of studies reported *in vitro* measurement of relaxation times, facilitating a comparison of the intrinsic properties of NMOCs without interference of biological tissues or physiological processes, potentially in a more controlled environment.^13^^,^^17^^,^^35^, ^36^, ^37^^,^^43^^,^^62^^,^^73^^,^^78^ Signal characteristics reported at a field strength of 1.5T were obtained for 17/31 (55 %) NMOCs, using either tabulated data or a graph plot digitiser, and are synthesised in Table 3. Although there is a large amount of missing and limited data, yerba mate exhibited the lowest reported T1 (155 ± 7 ms),^78^ followed by pineapple juice (185 ms–295.6 ms),^13^^,^^17^^,^^35^^,^^36^^,^^62^ with the longest T1 being water (2512 ms–4100 ms).^13^^,^^35^, ^36^, ^37^^,^^43^ The shortest reported T2 was pineapple juice (48 ms),^35^ however the range of pineapple juice reported T2 values was large owing to studies using different concentrations of pineapple juice. The longest T2 reported was water (2040 ms).^13^

**Table 3:** Relaxation times of NMOC media.

Non-medicinal contrast media	T1 values (ms)	T2 values (ms)	PD values (ms)	References
12 % corn oil emulsion	–	–	–	–
Acai juice	–	–	–	–
Apple and beetroot juice	1679	518	–	Arthurs et al. (2014)^35^
Apple juice	∼1753	∼543	–	Riordan et al., 2004)^13^
Black tea	–	–	–	–
Blackberry juice	831	103	–	Arthurs et al. (2014)^35^
Blackcurrant juice	2803	1480	–	Arthurs et al. (2014)^35^
Beet	–	–	–	
Blueberry and apple juice	∼359	∼72	–	Riordan et al., 2004)^13^
Blueberry juice	573	80	–	Arthurs et al. (2014)^35^
	356	64.1	–	Asbach et al. (2006)^36^
	–	86 ± 9	–	Renzulli et al. (2019)^17^
Cranberry juice	∼2235	∼800	–	Riordan et al., 2004)^13^
Date syrup	–	–	–	
Grape	–	–	–	
Grapefruit juice	∼2388	∼656	–	Riordan et al., 2004)^13^
Green tea	–	–	–	
Ice cream sediment	670	53	–	Bisset (1989)^37^
Infant feeding formula				
SMA gold	930	148	–	Arthurs et al. (2014)^35^
Humana 1 (Nestle)	1220	400	–	Balzarini et al. (1992)^73^
Nidina 1 (Nestle)	640	210	–	Balzarini et al. (1992)^73^
Isomil with iron	994	112	–	Bisset (1989)^37^
Similac with iron	580	90	–	
Similac with low iron	1438	103	–	
Similac 20	1484	104	1200	Gerscovich et al. (1990)^43^
Jasmine tea	–	–	–	
Medlar	–	–	–	
Milk				
Condensed milk	920	130		Balzarini et al. (1992)^73^
Whole milk	1739	92		Bisset (1989)^37^
Skimmed milk	1893	95	–	
	∼1804	∼144	–	Riordan et al., 2004)^13^
Orange juice	1796	440	–	Arthurs et al. (2014)^35^
	2720	690	–	Asbach et al. (2006)^36^
	∼2071	∼635	–	Riordan et al., 2004)^13^
Oolong tea	–	–	–	
Oxygen super saturated water	–	–	–	
Pineapple juice				
Brand 1	243	48	–	Arthurs et al. (2014)^35^
Brand 2	258	53	–	
	295.6	79.1	–	Asbach et al. (2006)^36^
Brand 1	–	60 ± 6	–	Renzulli et al. (2019)^17^
Brand 2	–	59 ± 6	–	
Brand 3	–	68 ± 7	–	
Brand 4	–	51 ± 5	–	
Brand 4 (75 % concentration)	–	59 ± 6	–	
Brand 4 (62.5 % concentration)	–	67 ± 7	–	
Brand 4 (50 % concentration)	–	80 ± 7	–	
Brand 4 (37.5 % concentration)	–	101 ± 10	–	
Brand 4 (25 % concentration)	–	140 ± 15	–	
100 % concentration	–	95	–	Renzulli et al. (2022)^62^
50 % concentration	–	172	–	
25 % concentration	–	330	–	
12.5 % concentration	–	747	–	
6.3 % concentration	–	1261	–	
	∼185	∼72	–	Riordan et al. (2004)^13^
Plum	–	–	–	
Prune juice	1245	324	–	Arthurs et al. (2014)^35^
	∼666	∼205	–	Riordan et al., 2004)^13^
Raspberry juice	1055	146	–	Arthurs et al. (2014)^35^
Roselle flower tea	–	–	–	–
Vanilla black tea	–	–	–	
Water	2831	1950	–	Arthurs et al. (2014)^35^
	3800	1800	–	Asbach et al. (2006)^36^
	2512	352	–	Bisset (1989)^37^
	2978	386	1082	Gerscovich et al. (1990)^43^
	∼4100	∼2040	–	Riordan et al., 2004)^13^
Yerba mate	155 ± 7	–	–	Nestle et al. (2004)^78^

NMOCs measured where the field strength was 1.5 T.

The ∼ symbol indicates the values were interpreted from a graph using a plot digitiser.

### MRI study preparation

Table 4 illustrates the various participant preparation methods reported in studies, segregated into those focused on tissue visualisation and those focused on motility. Four out of five studies reported a pre-imaging fasting requirement ranging between three to 12 h to try to ensure the stomach was empty. The use of erythromycin or metoclopramide in two papers was attributed to the known efficacy of these agents in enhancing gastric emptying, thereby retaining less contrast in the stomach. Of these two studies, Young et al. (2008) also referenced the potential for these agents to increase small bowel distension to improve visualisation.^47^^,^^57^ Other preparation methods included asking participants not to consume caffeine or tobacco during the day of the study (2/47, 4 %),^39^^,^^42^ and positioning of the participants in the right decubitus position for 15 min^66^ or 60 s prior to the examination to speed up gastric emptying (2/47, 4 %).^40^

**Table 4:** Study prior preparation protocols in visualisation and motility studies.

Prior preparation	Visualisation studies (42/47)	Motility studies (5/47)
Bowel motion stimulant	Two studies (5 %) reported the use of medicines to stimulate gastric emptying of oral contrasts, asking participants to consume either 10 mL of metoclopramide syrup (10 mg/mL) immediately prior to consumption of the oral contrast,^57^ or IV administration of erythromycin (100 mg) immediately after oral contrast ingestion.^47^	Zero
Antispasmodics	Six studies (15 %) reported the administration of hyoscine butylbromide (Buscopan) (three intramuscularly, two IV and one did not report the route of administration) with doses ranging between 10 mg and 40 mg.^40^^,^^45^^,^^46^^,^^52^^,^^53^^,^^58^ one study reported using either IV hyoscine (10 mg) or IV glucagon (0.25 mg).^63^	Zero
Fasting	Thirty-one studies (74 %) reported a fasting requirement. Mean and mode time of fasting was 6.9 h and 6 h, respectively, with a range of 3–12 h. Seven studies (17 %) required fasting for 12 h or overnight fasting^38^^,^^40^^,^^45^^,^^46^^,^^54^^,^^68^^,^^70^ and 11 studies (26 %) did not report any fasting requirements.^17^^,^^37^^,^^50^^,^^52^^,^^56^^,^^57^^,^^73^^,^^75^, ^76^, ^77^, ^78^	Four out of the five motility studies (80 %) reported a requirement for fasting.^44^^,^^59^^,^^60^^,^^64^^,^^71^ two of those studies required overnight fasting^60^^,^^71^ and the other two required fasting for 6 h prior to examination.^59^^,^^64^

### Oral contrast ingestion

The range of NMOC volume administered across all studies varied between 100 ml and 2000 ml, ingested between 0 and 180 min prior to MRI examination. For visualisation studies specifically, a slightly wider range of volumes of 100 ml–2000 ml were reported, compared to motility studies, where 500 ml–1200 ml were used. Both motility and visualisation themed studies reported a mode time of consumption prior to the MRI exam as 0 min. Data describing the average and range of volumes is presented in Supplementary material D, whilst a complete extraction of NMOC agent consumption, including the anatomical areas of interest, volume, and timing of administration of NMOCs is presented in Supplementary material E.

### Motion management

Twenty-two studies (47 %) utilised breath-hold to acquire the MRI images,^13^^,^^16^^,^^36^^,^^42^^,^^44^, ^45^, ^46^, ^47^, ^48^, ^49^, ^50^^,^^52^, ^53^, ^54^, ^55^^,^^59^^,^^64^^,^^67^^,^^69^^,^^71^^,^^74^^,^^77^ four studies did not,^35^^,^^40^^,^^56^^,^^65^ and 19 studies (40 %) did not state if imaging was acquired using breath hold or any other form of respiratory compensation.^17^^,^^37^, ^38^, ^39^^,^^41^^,^^43^^,^^51^^,^^57^^,^^61^^,^^62^^,^^66^^,^^68^^,^^70^^,^^72^^,^^73^^,^^75^^,^^76^^,^^78^ One study (2 %) reported that participants were asked to use ‘quiet breathing’ together with an abdominal compression band.^58^ One study (2 %) reported that participants were asked to voluntarily time their respiration with the scan; however, specific details on how this was achieved were not provided.^60^

### Impact on tissue conspicuity

NMOCs were administered to aid GI structure delineation or conspicuity in 42/47 studies (89 %). Of the remaining five studies, two utilised oral contrast as a mechanism to study motility and were not specifically measuring tissue conspicuity of the oral contrast.^44^^,^^71^ One further study did not measure tissue conspicuity but was focussed on the amount and timing of distention of the small bowel.^57^ Similarly, one enterography study also measured bowel distension as well as ‘image quality’ relating to the presence of artefacts with different oral contrast media but had no control and mostly compared distension of pineapple juice to different concentrations of mannitol.^63^ The focus of one other study was to study the fate of oxygen supersaturated water in the GI tract, but the authors did evaluate luminal signal intensity.^51^

### Participant experience

Twenty-four studies (51 %) did not report any data on participants' experiences. Ten studies (21 %) measured participant experience specifically related to oral contrasts.^13^^,^^16^^,^^41^^,^^47^^,^^48^^,^^57^^,^^63^^,^^71^^,^^72^^,^^74^ Of these, eight reported using questionnaires or scoring systems to assess participant experience.^16^^,^^41^^,^^47^^,^^48^^,^^57^^,^^63^^,^^71^^,^^74^ Only one of these studies^71^ provided references to the sources upon which their questionnaire was based, and just one study included the questionnaire itself in their appendix.^48^ The remaining 13 studies (28 %) briefly mentioned tolerability, focusing on side-effects.^37^^,^^40^^,^^45^^,^^46^^,^^49^^,^^54^^,^^65^, ^66^, ^67^^,^^69^^,^^70^^,^^73^^,^^75^

## Discussion

### Summary of evidence: mapping existing knowledge of NMOCs

This scoping review systematically mapped the literature regarding the utilisation of NMOCs used in upper-abdominal MRI studies. We identified 31 distinct NMOCs, two-thirds of which described an evaluation of potential tissue visualisation enhancement (89 %) with 11 % using NMOCs to observe motility. The data demonstrated a diverse range of NOMCs being explored in the literature, with water being the most frequently used and evaluated substance, followed by pineapple and blueberry juice. The justification for the investigation for some studies into the use of NMOCs was the lack of clinically available medicinal oral contrast agents. The heterogeneity of the studies, with different aims and methods, resulted in a rich diversity of NMOC uses. Although this diversity precluded direct comparisons of NMOC efficacy, potential applications of NMOCs in MR-guided radiotherapy have been identified, which is notably absent in the literature.

### Characteristics of NMOCs and their range of applications

The most common objectives of studies identified in this review were seeking to identify contrast agents for enterography or MRCP examinations by achieving adequate distension and/or signal suppression.^6^^,^^11^ There were some inconsistencies in the nomenclature of NMOC appearance in the published data (see Supplementary material F). There was consensus in terminology, where terms such as ‘positive’ or ‘negative’ were used to indicate luminal hyperintensity or hypointensity, respectively, but caution should be used where no relative reference has been provided. Ensuring clear and consistent terminology is used, potentially as described in Table 5, is recommended for future authors, although it is accepted signal appearance is relative and open to interpretation. To compare the appearance of contrast agents, the same magnet strength, sequences type and weighting should be used, as well as an appropriate reference such as abdominal muscle.^79^^,^^80^

**Table 5:** Luminal appearance of NMOCs based on T1 and T2 spin echo sequences in relation to abdominal muscle.

Relaxation times (T1 & T2)	T1w appearance		T2w appearance	
Shorter		Lighter/Hyperintense		Darker/Hypointense
Longer		Darker/Hypointense		Lighter/Hyperintense

### Identification of gaps in the evidence

The most significant gap identified by this review is the absence of studies examining the use of NMOCs in the context of MR-guided radiotherapy. Despite the promising characteristics of NMOCs in diagnostic MRI studies, their potential to improve radiotherapy planning and delivery remains unexplored. Distinguishing between different abdominal structures can be challenging,^4^ making this an important area for future targeted research, particularly given the growing integration of MRI into the radiotherapy pathway.

Although no empirical evaluations of NMOCs MR-guided radiotherapy were found in this scoping review, it is possible that clinical adoption within MR-guided radiotherapy has already taken place without substantial research to support it. For instance, a review by Boldrini et al. (2021) suggested that administering a glass of water prior to MR-guided radiotherapy may help visualise the stomach and duodenum due to the hyperintense signal of water.^81^ Similarly, an adaptive radiotherapy study using an MR-Linac to treat 30 patients with locally advanced pancreatic cancer described giving patients half a cup of water prior to treatment to better differentiate between the duodenum and gross tumour volume interface.^82^

The optimal selection of NMOC for the purposes of MR-guided radiotherapy may therefore depend on factors such as disease location, the intended benefit of MRI for organ delineation, and potentially, patient preference; an aspect not thoroughly examined by any of the research studies included in this review.

Optimising both patient comfort and treatment precision in radiotherapy is important, particularly in the context where patients often present with multiple comorbidities. This review revealed a lack of information regarding participant or patient experiences with NMOC ingestion. Studies frequently presented limited information on side-effects, palatability, tolerance, and compliance, with no data reported on quality of life. Despite 13 % of studies using a questionnaire to measure participant experience of ingesting oral contrast media, there was insufficient information reported to determine whether any of these instruments were validated. This lack of reporting limits the ability to accurately and reliably interpret and compare data on participant experience across studies,^83^ highlighting a potential gap in the evidence base. Woolen et al. (2017) explored the patient-centred value of oral contrast media used in CT, incorporating questions from the Testing Morbidities Index; validated measures of temporary health disutility.^84^^,^^85^ Their findings suggested that if oral contrast material offered a diagnostic benefit, most patients would prefer to ingest it rather than risk missing important findings.^84^ To facilitate future evaluations and comparisons of MRI oral contrast media, the use of validated questionnaires is recommended. Additionally, qualitative methods could provide further validation and offer a more holistic and nuanced understanding of patient perspectives.^86^ Although some authors claimed to have conducted ‘qualitative’ analysis, they appear to have been referring to quantitative measures of image quality, rather than employing true qualitative methodologies.^36^^,^^63^^,^^72^ Importantly, none of the studies included in this review utilised genuine qualitative methods in relation to NMOCs.

### Limitations

This review has several limitations. Although an information specialist supported the development of a search strategy, the literature search was conducted by one reviewer, with only 23 % of abstracts independently screened by a second. Relevant data may have therefore been missed. Furthermore, due to the possibility of introducing errors resulting from inaccurate translation, the search and exclusion strategy identified six full-text papers that were excluded because no English translation was available^29^, ^30^, ^31^, ^32^, ^33^, ^34^ raising the possibility that key information may have been lost.

The heterogeneity of studies and their methods prevented a meta-analysis of multiple outcomes related to NMOCs, and this was deemed outside of the scope of this review. There was also inconsistency in study methods when comparing image quality, and limited information on the clinical significance of reported results. Whilst extracting more data on tolerability and experience was intended, this information was either only briefly covered or entirely absent from most studies.

Given this scoping review aimed to develop a comprehensive overview of the evidence surrounding NMOCs rather than a complete synthesis of outcomes data, a methodological appraisal or risk of bias assessment of studies was not conducted. Although this approach is in line with supporting guidance for scoping reviews, some data, such as the likely appearance and reported relaxometry of specific NMOCs was synthesised to facilitate further research. However, these results should be interpreted with caution, as they lack the quality and reliability evaluations that ensure greater validity.^87^^,^^88^

Despite these limitations, this scoping review is the first to systematically summarise the properties of NMOCs and highlights their potential use in MR-guided radiotherapy. By providing a comprehensive overview of the current evidence, this review has established a foundation for future research and offers insights into the emerging applications of NMOCs in radiotherapy planning and delivery.

## Conclusion

NMOCs have been extensively used in diagnostic MRI to enhance GI visualisation and assess motility, with this review identifying 31 distinct NMOCs evaluated in the literature, primarily used to aid tissue visualisation. Despite their promising characteristics, such as the potential to improve tissue conspicuity and having fewer side effects than their medicinal counterparts, their application in MR-guided radiotherapy remains unexplored. Further research is needed to evaluate NMOCs in this context, considering imaging requirements, as well as patient experience and acceptability.

## Conflict of interest statement

The authors declare that they have no conflicts of interest.
