# Supplementary material for: Non-medicinal oral contrast in upper abdominal MRI for MR-guided radiotherapy: A scoping review
Source: Radiography (Lond). 2025 Mar;31(2):None. doi: 10.1016/j.radi.2025.01.003 (PMC11904122; doi:10.1016/j.radi.2025.01.003)
Supplement: Multimedia component 1 [file mmc1.docx]

Supplementary material

Supplementary material A: Search strategy

**CAB Abstracts**

**Embase**

**Ovid MEDLINE(R) ALL**

1 exp abdomen/

2 exp gastrointestinal tract/

3 exp liver/

4 exp pancreas/

5 exp stomach/

6 (lumen or duodenum or bowel or bile duct or biliary or hepatobiliary or enteric or gastric or abdom*).tw.

7 1 or 2 or 3 or 4 or 5 or 6

8 magnetic resonance.tw.

9 MR.tw.

10 MRI.tw.

11 8 or 9 or 10

12 exp contrast media/

13 oral.tw.

14 12 and 13

15 oral contrast.tw.

16 gastrointestinal contrast.tw.

17 enteric contrast.tw.

18 pineapple.tw.

19 blueberry.tw.

20 milk*.tw.

21 water.tw.

22 15 or 16 or 17 or 18 or 19 or 20 or 21

23 14 or 22

24 exp animals/ not humans.sh.

25 7 and 11 and 23

26 25 not 24

**EBSCO CINAHL**

( ( MH "Abdomen+" OR "GI" OR (MH "Liver+") OR (MH "Pancreas+") OR (MH "Stomach+") OR ( TX lumen OR TX duodenum OR TX bowel OR TX bile duct OR TX biliary OR TX hepatobiliary OR TX enteric OR TX gastric OR TX abdom* ) ) AND ( (MH "Diagnostic Imaging+") OR TX magnetic resonance OR TX MRI ) AND ( ( TX oral contrast OR TX pineapple OR TX blueberry OR TX milk OR TX water ) AND ( (MH "Contrast Media+") AND TX oral ) ) ) NOT ( (MH animals+ OR MH (animal studies) OR TI (animal model*) ) NOT MH (human) )

**ProQuest Dissertations and Theses**

("oral contrast" OR "enteric contrast" OR "gastrointestinal contrast" OR pineapple OR milk* OR blueberry OR water) AND (gastrointestinal OR liver OR pancreas OR stomach OR lumen OR duodenum OR bowel OR "bile duct" OR biliary OR hepatobiliary OR enteric OR gastric OR abdom*) AND (MRI OR MR OR "magnetic resonance") AND subt.exact("medical imaging")

**Google Scholar**

(("oral contrast" AND natural)) AND (gastrointestinal OR liver OR pancreas OR stomach OR lumen OR duodenum OR bowel OR "bile duct" OR biliary OR hepatobiliary OR enteric OR gastric OR abdom*) AND (MRI OR magnetic resonance)

Supplementary material B: Characteristics of the sources of evidence including funding and possible conflict of interest statements

| Study ID | Title | Type of oral contrasts tested | Type of publication | Year of publication | Method | Participant types | Aim of study | Study funding | Possible conflicts of interest | MRI field strength |
| --- | --- | --- | --- | --- | --- | --- | --- | --- | --- | --- |
| Arruda-Sanchez 2006 | Preliminary results from clinical application of a natural oral contrast agent in magnetic resonance imaging (MRI) of the gastrointestinal (GI) system | Milk; Other: Acai (Euterpe Oleracea) | Conference abstract | 2005 | In vivo | Healthy volunteers; Patients | To investigate the feasibility of using a natural solution as a gastrointestinal oral negative contrast agent to null the bowel signal during MRCP. | ‘This work was supported in part by the CNPq and CAPES.' | None stated | 1.5T |
| Arthurs 2014 | Interactive neonatal gastrointestinal magnetic resonance imaging using fruit juice as an oral contrast media | Pineapple juice; Other: 12 different measured in vitro first: tap water, infant formula (SMA gold) Iopamidol (Gastromiro), and 9 commercially readily available fruit juices - pineapple (concentrate and non), orange juice, apple and beetroot juice, prune juice, blackcurrant juice, blueberry juice, raspberry juice, blackberry juice.   In-vivo: Only pineapple studied in vivo. | Full paper | 2014 | In vitro; In vivo | Healthy volunteers | To evaluate the in vitro properties of fruit juice and then perform in vivo evaluation with an interactive inversion recovery (IR) MR pulse sequence to visualise the gastrointestinal tract. | ‘Medical Research Council/Royal College of Radiologist Clinical Research Training Fellowship. None of the funding bodies had any role in the analysis of data, results, or conclusions of the study.' | The authors declared no conflict of interest. | 1.5T |
| Asbach 2006 | Cine magnetic resonance imaging of the small bowel: comparison of different oral contrast media | Pineapple juice; Blueberry juice; Water; Other: Orange juice | Full paper | 2006 | In vitro; In vivo | Healthy volunteers; Patients | To evaluate several substances impact on small bowel distension and contrast on balanced steady-state free precession (bSSFP) cine magnetic resonance (MR) images. | Not stated | Not stated | 1.5T |
| Balzarini 1992 | Magnetic resonance imaging of the gastrointestinal tract: investigation of baby milk as a low cost contrast medium | Other: Baby milk (Nidina 1) | Full paper | 1992 | In vitro; In vivo | Patients | The aim of this study was to investigate the origin of the different effects of various milk specimens on imaging. Also to evaluate possible roles as contrast mediums in clinical practice. | Not stated | Not stated | 1.5T |
| Bisset 1989 | Evaluation of potential practical oral contrast agents for pediatric magnetic resonance imaging. Preliminary observations | Milk; Other: Following the in vitro testing, Similac with standard iron and whole milk were given to 4 normal fasted volunteers ranging in age from 10-28 years.   'In vitro 60cc of: 1) Chloral hydrate suspension (commonly used sedative) in a concentration of I00 mg/cc 2) Similac with low iron (1.5 mg elemental iron/liter) (Ross Laboratories, Co- lumbus, Ohio) 3) Similac with standard iron (12 mg elemental iron/liter) (Ross Laboratories, Columbus, Ohio) 4) Isomil with standard iron (12 mg elemental iron/liter) (Ross Laboratories, Columbus, Ohio) 5) Whole milk 6) Skim milk 7) Breast milk 8) Ice cream sediment (inert plastic used as hardening agent layered on top) 9) Tap water.' | Full paper | 1989 | In vitro; In vivo | Healthy volunteers | Perform in vitro and in vivo investigation of several practical, low cost, high signal intensity contrast agents. | Not stated | Not stated | 1.5T |
| Cordova-Fraga 2004 | Euterpe Oleracea (Acai) as an alternative oral contrast agent in MRI of the gastrointestinal system: preliminary results | Water; Other: Acai followed the water so they have scans of empty stomach (air, then water then acai. | Full paper | 2004 | In vitro; In vivo | Healthy volunteers | Test a new oral contrast, readily available in Brazil. | ‘This work was funded in part by Fapesp, CNPq, CAPESand Conacyt.’ | Not stated | 1.5T |
| Cordova-Fraga 2012 | Medlar (Achras sapota L.) as oral contrast agent for MRI of the gastrointestinal tract | Other: Achras sapotaL. (common medlar) | Full paper | 2012 | In vitro; In vivo | Healthy volunteers | Present an alternative fruit Achras sapota L (common medlar) as a clinical oral contrast agent for MRI of the GI system. | ‘The Support to Research and Postgrad Bureau’, (DAIP) under grant number 0017/2011. | Not stated | 1.5T |
| Cronin 2009 | Hypotonic MR duodenography with water ingestion alone: feasibility and technique | Water | Full paper | 2009 | In vivo | Healthy volunteers | The purpose of this study was to 1) describe and assess the feasibility of performing a per-oral, single contrast, hypotonic magnetic resonance (MR) duodenography technique and 2) assess the efficacy of intravenous Buscopan in facilitating duodenal distension at cross-sectional MR imaging. | Not stated | Not stated | 1.5T |
| Elsayed 2015 | Effectiveness of natural oral contrast agents in magnetic resonance imaging of the bowel | Pineapple juice; Milk; Water | Full paper | 2015 | In vivo | Healthy volunteers | The purpose was to find an oral contrast agent that can be utilised in MRI of the small bowel 'and fulfill the following criteria: natural, with no or minimal side effects, non-expensive, causes the maximum bowel distension and has the best imaging quality with no artifacts.' | Not stated | Not stated | 1.5T |
| Espinosa 2006 | Blackberry (Rubus spp.): a pH-dependent oral contrast medium for gastrointestinal tract images by magnetic resonance imaging | Other: Blackberry (Rubus spp.) | Full paper | 2006 | In vitro; In vivo | Healthy volunteers | The characterisation of magnetic properties of seven different fruits and T1- and T2-weighted MR images of a phantom and a subject. | ‘The authors acknowledge the financial support of CONACyT under grant number 38749-E. Support for MR resources came from The Gonzalo Rio Arronte Foundation. FAB acknowledges the financial support of PAPIIT-DGAPAIN214304.’ | ‘FAB acknowledges the financial support of PAPIIT-DGAPAIN214304.’ | Other: 1.0 T |
| Evans 1993 | Prolonged monitoring of the upper gastrointestinal tract using echo planar magnetic resonance imaging | Water; Other: Egg and mayonnaise sandwich with an additional 500ml water to observe fed state. | Full paper | 1993 | In vivo | Healthy volunteers | Attempt to 'visualise the two major physiological states of upper gastrointestinal motility: the fasting state and the fed state.' | ‘The Medical Research Council, the Department of Health of Great Britain, and the British Heart Foundation, all financially support the Echo-Planar imaging programme in Nottingham. The Deutsche Forschungsgemeinschaft, the Science and Engineering Research Council, Analogic Inc, and Oxford Magnet Technology are acknowledged for contributions towards salaries and hardware.’ | Not stated | Other: Custom 0.52 T corresponding to a resonance frequency for protons of 22 MHz. |
| Gerscovich 1990 | The rediscovery of infant feeding formula with magnetic resonance imaging | Other: Infant feeding formula | Full paper | 1990 | In vivo; in vitro | Healthy volunteers; Patients | Describe a multipurpose oral agent that provides opacification of the gastrointestinal tract during paediatric MRI. | Not stated | Not stated | Other: Adult = 0.5 T and 1.5T Infants = 0.5T *2 and 1.5T *4 (only six provided in the table - two excluded). |
| Ghanaati 2011 | Improvement of MR cholangiopancreatography (MRCP) images after black tea consumption | Other: Black tea | Full paper | 2011 | In vivo; in vitro | Patients | Evaluation of the efficacy of black tea as a negative oral contrast agent in MRCP. | Not stated | Not stated | 1.5T |
| Govindarajan 2014 | Evaluation of date syrup as an oral negative contrast agent for MRCP | Milk; Other: Date syrup | Full paper | 2014 | In vitro; In vivo | Patients | Compare the in vitro effect of date syrup with that of other contrast agents in T2w and MRCP sequences, quantify the iron concentration, and evaluate the resultant quality of MRCP images. | Not stated | Not stated | 1.5T |
| Hiraishi 1995 | Blueberry juice: preliminary evaluation as an oral contrast agent in gastrointestinal MR imaging | Blueberry juice; Other | Full paper | 1995 | In vitro; In vivo | Healthy volunteers | Evaluate the use of blueberry juice as an oral contrast agent in MRI. | Not stated | Not stated | 1.5T |
| Hosseini 2021 | Quantification of Gastric Contractions Using MRI with a Natural Contrast Agent | Pineapple juice | Full paper | 2021 | In vivo | Healthy volunteers | No aim actually stated, however, they were aiming to investigate using MRI with pineapple juice as a contrast agent to help estimate the center line of the stomach, and quantify contraction speeds. | ‘Research supported, in part, by a University of Auckland Doctoral Scholarship, Riddet Institute, Medical Technologies Centre of Research Excellence (MedTech CoRE) and the Health Research Council of New Zealand.’ | Not stated | Other: 1.5 T and 3T |
| Inoue 2018 | Acceleration of small bowel motility after oral administration of dai-kenchu-to (TJ-100) assessed by cine magnetic resonance imaging | Water | Full paper | 2018 | In vivo | Healthy volunteers | To evaluate the effect of TJ100 on small intestinal movements using cine MRI in healthy volunteers. | None | The authors declared no conflict of interest. | 1.5T |
| Karantanas 2000 | Blueberry juice used per os in upper abdominal MR imaging: composition and initial clinical data | Blueberry juice | Full paper | 2000 | In vitro; In vivo | Healthy volunteers; Patients | Evaluate blueberry juice as a positive and negative agent and also quantify the paramagnetic contents. | Not stated | Not stated | Other: 1 T |
| Kim 2000 | MRI in staging advanced gastric cancer: is it useful compared with spiral CT? | Water | Full paper | 2000 | In vivo | Patients | Compare MRI to CT for staging of advanced gastric cancer. | Not stated | Not stated | Other: 1.0 T |
| Kim 2000 | MR imaging of advanced gastric cancer: comparison of various MR pulse sequences using water and gadopentetate dimeglumine as oral contrast agents | Water; Other: gadopentetate dimeglumine | Full paper | 2000 | In vivo | Patients | Evaluate different MRI techniques and the 'usefulness' of water and Gd-GTPA as oral contrast agents in advanced gastric cancer cases. | Not stated | Not stated | Other: 1.0 T |
| Krishnasamy 2020 | Processing Apples to Puree or Juice Speeds Gastric Emptying and Reduces Postprandial Intestinal Volumes and Satiety in Healthy Adults | Other: Apple, apple puree or apple juice | Full paper | 2020 | In vivo | Healthy volunteers | Assess 'the intragastric processing of apple preparations and the associated small and large bowel contents using MRI.' | ‘Supported by a research grant from the Ministry of Higher Education Malaysia and the University of Nottingham.’ | ‘Declared:  Author disclosures: RCS has received research grant support from Zespri Inter-national and Ironwood Pharmaceuticals Inc. MCEL is a coinventor of a mobile application relating to the low fermentable, oligo-di-and monosaccharides and polyols (FODMAP) diet diet. The authors report no conflicts of interest.’ | 1.5T |
| Kuehle 2006 | Hydro-MRI of the small bowel: effect of contrast volume, timing of contrast administration, and data acquisition on bowel distention | Water; Other: Water was one of four agents tested. The other three were 0.2% locust bean gum plus 2.5% mannitol, VoLumen containing 2.0% sorbitol, VoLumen containing 1.4% sorbitol. | Full paper | 2006 | In vivo | Healthy volunteers | Assess oral contrast agents, volumes of the agents, and time points regarding small-bowel distention and patient acceptance.' | Not stated | Not stated | 1.5T |
| Lam 2007 | MRI: imaging of stomach | Blueberry juice; Water; Other: 75% barium (the blueberry juice had a concentration of 70%) | Full paper | 2007 | In vivo | Healthy volunteers | To determine the optimal MRI bowel preparation regime for visualisation of the stomach anatomy.' | Not stated | Not stated | 1.5T |
| Lauenstein 2003 | Optimization of oral contrast agents for MR imaging of the small bowel | Water; Other: and water in combination with mannitol, a bulk fiber laxative, locust bean gum, and a combination of mannitol and locust bean gum. | Full paper | 2003 | In vivo | Healthy volunteers | To ‘assess the effect on small-bowel distention of osmotic and non-osmotic additives to water as contrast agents for MR imaging.' | Not stated | Not stated | 1.5T |
| Lomas 1999 | Small bowel MRI using water as a contrast medium | Water | Short communication | 1999 | In vivo | Healthy volunteers | Determine the feasibility of using rapid heavily T2w techniques combined with oral water loading for imaging of the luminal small bowel in volunteers. | Not stated. | Not stated | 1.5T |
| Minowa 1999 | MR imaging of the small bowel using water as a contrast agent in a preliminary study with healthy volunteers | Water | Full paper | 1999 | In vivo | Healthy volunteers | To introduce and evaluate a new MR imaging technique of combining water as an oral contrast agent with heavily T2 weighted images using a fast advanced spin echo sequence for the visualisation of small bowel lumen and folds. | Not stated | Not stated | 1.5T |
| Mohabir 2020 | The efficacy of pineapple juice as a negative oral contrast agent in magnetic resonance cholangiopancreatography | Pineapple juice | Full paper | 2020 | In vivo | Patients | Assess the role of ‘off-the-shelf’ pineapple juice in an easily consumable quantity (250ml) as a negative oral contrast agent for MRCP. | None | The authors declared no conflict of interest. | 1.5T |
| Nestle 2004 | In vivo observation of oxygen-supersaturated water in the human mouth and stomach | Other: Oxygen super saturated water | Full paper | 2004 | In vivo | Healthy volunteers | Observe the behaviour of oxygen super saturated water in the oral cavity and stomach on MRI compared with normal water. | ‘N.N. furthermore acknowledges fundingunder DFG grant BA 1592/1-1 at the TU Munich.’ | Not stated | 1.5T |
| Nestle 2006 | Oral magnetic resonance imaging contrast agent based on Ilex paraguayensis herbal extract | Yerba mate | Full paper | 2006 | In vitro; In vivo | Healthy volunteers; Patients | Demonstrate the potential of yerba mate as an oral contrast agent. | Not stated | None stated | 1.5T |
| Papanikolaou 2000 | MR cholangiopancreatography before and after oral blueberry juice administration | Blueberry juice | Full paper | 2000 | In vivo | Patients | Evaluate blueberry juice as a negative oral contrast agent for the gastrointestinal tract in MRCP studies. | Not stated | Not stated | 1.5T |
| Pinho 2019 | Image Quality in Magnetic Resonance cholangiopancreatography exams: study between aÃ§ai juice and a manufactured contrast agent | Other: Acai juice and manufactured iron oxide-based contrast | Full paper | 2019 | In vivo | Patients | To assess image quality obtained with the administration of acai juice as compared to a manufactured standard iron oxide-based contrast employed as negative oral agents in MRCP. | ‘This research was partially funded by Funda Araucaria from Paranai State (Brazil), project 355/2012.’ | The authors declared no conflict of interest. | 1.5T |
| Renzulli 2019 | What is the best fruit juice to use as a negative oral contrast agent in magnetic resonance cholangiopancreatography? | Pineapple juice; Blueberry juice; Other: 4 pineapple juices and one blueberry juice. | Full paper | 2019 | In vitro; In vivo | Healthy volunteers | Identify the best fruit juice to use as an oral negative contrast agent for MRCP and determine correspondingly optimal parameters. | Not stated | The authors declared no conflict of interest. | 1.5T |
| Renzulli 2022 | Optimization of pineapple juice amount used as a negative oral contrast agent in magnetic resonance cholangiopancreatography | Pineapple juice | Full paper | 2022 | In vitro; In vivo | Patients | Assess the potential variability of the Mn2+ content in pineapple juice of the same commercially available brand produced in different years and identify the optimal concentration of Mn2+ and the correct amount of pineapple juice to be orally administered to fasting patients prior to MRCP to suppress the gastroduodenal liquid signal. | Not stated | The authors declared no conflict of interest. | 1.5T |
| Riordan 2004 | Pineapple juice as a negative oral contrast agent in magnetic resonance cholangiopancreatography: a preliminary evaluation | Pineapple juice; Blueberry juice; Milk; Water; Other: Orange juice, grapefruit juice, apple juice, prune juice, cranberry juice, blueberry and apple juice combined, 'standard radiological contrast agents' - barium EZ Cat 2% w/v, dilute barium (50:50 with water), concentrated gastrografin, ferumoxsil, and water as a control. | Full paper | 2004 | In vitro; In vivo | Healthy volunteers | Evaluate pineapple juice as an oral negative contrast agent in MRCP. | Not stated | Not stated | 1.5T |
| Sanchez 2009 | Clinical feasibility of Acai (Euterpe oleracea) pulp as an oral contrast agent for magnetic resonance cholangiopancreatography | Other: Acai | Full paper | 2009 | In vivo | Healthy volunteers; Patients | Evaluate the effectiveness of the Acai as a negative oral contrast to routine MRCP. | ‘This work was funded by CNPq, CAPES, and FAPESP.’ | Not stated | 1.5T |
| Scheibl 2005 | Magnetic resonance imaging gastrography: evaluation of the dark lumen technique compared with conventional gastroscopy in patients with malignant gastric disease | Water | Full paper | 2005 | In vivo | Patients | Demonstrate the feasibility of depicting gastric tumors using the dark lumen technique. | Not stated | Not stated | 1.5T |
| Sohn 2000 | Comparing MR imaging and CT in the staging of gastric carcinoma | Water; Other: Effervescent granules | Full paper | 2000 | In vivo | Patients | To assess the usefulness of breath-hold 2D fast low-angle shot and T2-weighted turbo spin-echo fast MR imaging compared with helical CT in the staging of gastric carcinoma | Not stated | Not stated | 1.5T |
| Sood 2002 | Small bowel MRI: comparison of a polyethylene glycol preparation and water as oral contrast media | Water; Other: polyethylene glycol | Full paper | 2002 | In vivo | Healthy volunteers | To compare water and a polyethylene glycol as potential oral contrast media for small bowel MRI. | ‘RRS is in receipt of a Cambridge University Nehru Scholarship.’Smith's Charity; Contract grant sponsor: Fund for Addenbrooke's also stated. | Not stated | Other: Not stated |
| Tart 1991 | Enteric MRI contrast agents: comparative study of five potential agents in humans | Other: 12% corn oil emulsion, 1 mM Geritol, Kaolin-pectin, single contrast oral barium sulfate, and effervescent granules. 'The corn oil emulsion was 12% corn oil emulsion, prepared by blending 473 ml of homogenized milk, three scoops of Breyers vanilla ice cream (Kraft, Inc., Glenview, IL) and 60 ml of Mazola corn syrup (CPC International, Englewoods Cliffs, NJ).' | Full paper | 1991 | In vivo | Healthy volunteers | To objectively compare several available potential enteric MR contrast agents. | Not stated | Not stated | 1.5T |
| Utami 2021 | Jasmine tea as a negative oral contrast agent in magnetic resonance cholangiopancreatography (MRCP) | Other: Jasmine tea | Full paper | 2021 | In vitro; In vivo | Healthy volunteers | To find a 'safe, affordable, and easily accessible alternative to natural oral negative MRCP.' Also to determine an optimal consumption to scan start time of the alternative negative oral contrast agent. | Not stated | Not stated | Other: Not stated |
| Varavithya 2005 | The efficacy of roselle (Hibicus sabdariffa Linn.) flower tea as oral negative contrast agent for MRCP study | Other: Roselle flower tea (Hibicus Sabdariffa Linn.) | Full paper | 2005 | In vitro; In vivo | Healthy volunteers; Patients; Other: Phantom, then healthy volunteers, then patient clinical study | Investigate the efficacy of roselle flower tea as an oral negative contrast agent for MRCP study. | Not state | Not stated | 1.5T |
| Videira 2016 | Magnetic resonance cholangiopancreatography applying a natural negative oral contrast: black tea | Other: Black tea | Conference abstract | 2016 | In vivo | Healthy volunteers | To optimise their MRCP acquisition protocol applying a natural negative oral contrast agent. | Not stated | Not stated | Other: Not stated |
| Wesbey 1985 | Dilute oral iron solutions as gastrointestinal contrast agents for magnetic resonance imaging; initial clinical experience | Water; Other: ferric ammonium citrate compared with water | Full paper | 1985 | In vivo | Patients | Compare the efficacy of ferric ammonium citrate and water in abdominal MRI. | ‘Supported in part by NIH Grant #ROl AM 31937-02 from the National Institutes of Arthritis, Diabetes and Kidney Diseases and by Schearing A.G. Berlin. ‘ | Not stated | Other: 0.35 T |
| Wright 1999 | Validation of antroduodenal motility measurements made by echo-planar magnetic resonance imaging | Water; Other: 500mL porridge | Full paper | 1999 | In vivo | Healthy volunteers | To compare antroduodenal motility measurements by simultaneous perfused tube manometry and echo planar imaging. | ‘Medical Research Council for the funding of the echo-planar imaging programme.’ | Not stated | Other: 0.5T |
| Young 2008 | Head-to-head comparison of oral contrast agents for cross-sectional enterography: small bowel distention, timing, and side effects | Water; Other: neutral oral contrast agents studied were water, methylcellulose, polyethylene glycol, and a low-concentration barium. | Full paper | 2008 | In vivo | Healthy volunteers | To determine optimal timing between ingestion and scanning of four commercial oral contrast agents and identify frequency and severity of side effects. | Not stated | ‘One co-author (J.G.F.) received grant support from one of the manufacturers of one of the oral agents examined herein and consequently did not participate in any quantitative or qualitative assessment of small bowel distention.’ | 1.5T |
| Zhong 2005 | Preoperative diagnosis of gastric cancer using 2-D magnetic resonance imaging with 3-D reconstruction techniques | Water | Full paper | 2005 | In vivo | Patients | Investigate the value of preoperative gastric cancer diagnosis using 2D MRI with water and 3D post-processing techniques for MR hydrography. | ‘This study was supported by Renji Hospital Scientific Research Fund.’ | Not stated. | Other: 1.0T |
| Zulkifle 2023 | Comparison of pineapple juice and mannitol as oral contrast agents for magnetic resonance enterography | Pineapple juice; Other: mannitol 6.7% and 3.3% | Full paper | 2023 | In vivo | Patients | Compare bowel distension and image quality between two mannitol concentrations and pineapple juice for enterography. | None | The authors declared no conflict of interest. | Other: T Verio and 1.5T Verio |

Supplementary material C: Study designs

| Study design | Healthy volunteers | Healthy volunteer and Patients | Patients | Grand Total |
| --- | --- | --- | --- | --- |
| Controlled before-after study |  | 1 | 2 | 3 |
| Other: Retrospective uncontrolled study |  | 1 |  | 1 |
| Other: Combined uncontrolled before after study for two of the volunteers (5 min and 40 min after ingestion and uncontrolled study for two other volunteers. | 1 |  |  | 1 |
| Randomised controlled trial | 1 |  |  | 1 |
| Uncontrolled before-after study | 20 | 3 | 13 | 36 |
| Uncontrolled study (no comparator) | 3 | 1 | 1 | 5 |
| Grand Total | **25** | **6** | **15** | **4** |

Supplementary material D: Oral contrast ingestion volume and timing overview

| Oral contrast administration | Visualisation studies (41/47) | Motility studies (5/47) |
| --- | --- | --- |
| Mean total volume | 657ml | 760ml |
| Mode total volume | 1000ml | 500ml |
| Volume range | 100ml – 2000ml | 500ml – 1200ml |
| Mean time of consumption prior to the MRI exam | 29.7 min | 6.7 min |
| Mode time of consumption prior to the MRI exam | 0 | 0 |
| Range of consumption time prior to MRI exam | 0 – 180 min | 0 – 20 min |

Supplementary material E: NMOC ingestion details by anatomical site of interest

| Anatomical site of interest | Volume | Timing of administration prior to MRI | Notes | Reference |
| --- | --- | --- | --- | --- |
| Mouth and stomach | 700 ml of oxygen supersaturated water and 1000 ml water. | Immediately before and 30 minutes before MRI |  | Nestle et al 2004^51^ |
| Stomach | 200 ml water followed by 200ml of Acai pulp | Not stated |  | Arruda-Sanchez (2006)^68^ |
|  | 200 ml water followed by MRI followed by 200 ml of Acai | Not stated |  | Cordova-Fraga (2004)^38^ |
|  | 400 ml: 200 ml water followed by 200 ml of Acai | Not stated. |  | Cordova-Fraga et al (2004)^38^ |
|  | 200 ml water and 200mg of Achras sapota L. pulp | Not stated |  | Cordova-Fraga et al (2012)^39^ |
|  | 300 ml water + blended plum | Five minutes before |  | Espinosa et al (2006)^42^ |
|  | 300-500 ml | Immediately before |  | Hosseini et al (2021)^64^ |
|  | 900 ml water followed by exam and the additional 100 ml of Gd-DPTA with 15g of mannitol | Before | Additional 100 ml ingested followed by three turnings on the exam table. | Kim et al (2000)^46^ |
|  | 1000 ml | Immediately before |  | Kim et al (2000)^45^ |
|  | ‘At least 500 ml’ and ‘asked to drink as much as possible or until they felt extremely full.’ | Within 5 minutes of oral ingestion |  | Lam et al (2007)^48^ |
|  | 1000 ml | 1 hour before MRI |  | Nestle et al 2006^78^ |
|  | 1000 ml | 15 minutes before |  | Scheibl et al (2005)^52^ |
|  | 500 ml water (n=21) or effervescent granules (n=9) | Not stated |  | Sohn et al (2000)^53^ |
|  | 500 ml | Immediately before |  | Wright (1999)^60^ |
|  | 800 ml-1000mL | Not stated |  | Zhong et al (2005)^58^ |
| Duodenum | 1000 ml | Immediately before | Participants asked to lay on their right side for 60 seconds to encourage contrast to pass form the stomach to the duodenum. | Cronin et al (2009)^40^ |
| Stomach and duodenum | 200 ml (adults) and 20 ml (neonate) | 10 minutes before exam |  | Arthurs et al (2014)^35^ |
|  | 400 ml divided into two equal parts | Two 200 ml parts consumed with an interval of 20 minutes before the exam |  | Balzarini et al (1992)^73^ |
|  | 1000 ml water in a fasting state image and a separate image session following consumption of an egg and mayonnaise sandwich plus 500 ml of water. | MRI immediately after consumption of oral contrasts |  | Evans et al (1993)^59^ |
| Stomach; Duodenum; Other | 500 ml | 40 minutes before MRI. | Stomach, duodenum, adjacent viscera, and distal small bowel to be differentiated from the colon. | Bisset (1989)^37^ |
|  | 250 – 900 ml | 30 minutes – 2.5 hours prior to MRI | Stomach; 'Small bowel' and ‘colon’. | Gerscovich et al (1990)^43^ |
|  | 300 ml | MRI ‘…before and after 5 and 15 min ingestion of 300 ml of tea infusion…’ | Stomach, duodenum, deptiction of the gall bladder, cystic duct, proximal and distal parts of the common bile duct, intrahepatic ducts, ampulla of Vate, main pancreatic duct, signal loss of the stomach and three distinct segments of the duodenum. | Ghanaati et al (2011)^75^ |
|  | 100 ml | 30 minutes before MRI. | Stomach; Duodenum; Other: Signal intensity: ‘The impedance of visibility of the pancreaticobiliary tree from the stomach or duodenum.’ | Govindarajan (2014)^72^ |
|  | ‘300-500 ml’ | MRI ‘just after they had ingested the blueberry juice.’ | Stomach, duodenum, ‘small bowel’ and pancreas. | Hiraishi et al (1995)^65^ |
|  | 1200 ml tap water | Not specifically stated – after ‘each volunteer drank… and underwent MRI examinations.’ | Stomach, duodenum, jejunum, ileum, ‘entire abdomen’. | Inoue et al (2018)^44^ |
|  | 430ml total over 60 minutes. | 150ml 60 minutes before MRI; 150ml 30 minutes before MRI; 130ml just before MRI. | Stomach, duodenum, ‘proximal small intestine’ | Karantanas et al (2000)^66^ |
|  | 600 ml. ‘Whole apples were served with 173 mL water, apple puree with 224 mL water, and apple juice with 260 mL water to obtain a final volume of 600 mL.’ | Either whole apples or apple puree or apple juice and completed consumption within 20 min. Although most subjects consumed the juice within 5-10 min, it took them around 15 to 20 min to consume both puree and apples. | Stomach; 'Small bowel' and ‘colon’. | Krishnasamy et al 2020^71^ |
|  | 250 ml | ‘After oral contrast.’ | Visibility of the common bile duct (CBD) and the main pancreatic duct (MPD) | Mohabir et al (2020)^61^ |
|  | 200 ml | One dose was given after the patient responded the anamnesis, and another was given 10 minutes later. | Stomach, duodenum, biliary tract | Pinho et al (2019)^69^ |
|  | 150 ml | 30 minutes before MRI. | Stomach, duodenum; Other: ‘the value of SI of a region of interest (ROI) of 0.5 cm3 was measured in 3 different sites: in the second portion of the duodenal lumen, in the region of the gastric fundus lumen and in correspondence of a reference liquid, meaning the location where the highest liquid intensity could be measured for each patient (such as gallbladder or biliary tract fluid, cerebrospinal fluid or renal cyst content).’ | Renzulli et al (2022)^62^ |
|  | 400 ml | 15 minutes and 30 minutes after oral contrast ingestion. | Various pancreaticobiliary tree segments | Riordan et al (2004)^13^ |
|  | 200 ml | Not stated: ‘… after the ingestion of Acai.’ | Stomach, duodenum, common bile duct, cystic duct, ampulla of Vater, and pancreatic duct | Sanchez et al (2009)^70^ |
|  | 300 ml | Before scan, and at 3-, 6-, and 9-minute time variations. | Stomach; Duodenum; Other: ‘Gall bladder (GB), cystic duct (CD), common bile duct (CBD), common hepatic duct (CHD), intrahepatic duct(IHD), ampulla vateri (AV) and main pancreatic duct(MPD) as well as the intensity of the gastric and duodenal signals was done...’ | Utami et al (2021)^55^ |
|  | 500 ml water (control) consumed at different time points. | 250 ml consumed 20-40 minutes before imaging with the patient upright, and another 250 ml consumed 5-10 minutes before imaging. | Stomach, duodenum, jejunum, ileum | Wesbey (1985)^56^ |
|  | 2000 ml divided and consumed over time | 500 ml at 0 min, two additional 500 ml aliquots of the oral contrast agent were taken at 5 and 10 minutes after the initiation of ingestion, with two smaller aliquots of 250 ml ingested at 25 and 35 minutes. | Stomach, duodenum, jejunum, ileum, ‘whole GI tract’ | Young et al (2008)^57^ |
| Duodenum; Other (Jejunum, Ileum including terminal ileum) | 1500ml divided into three doses over time | 600ml 60 min before the scan, 600ml 30 min before the scan, and 300ml just prior to the scan. | Duodenum, jejunum, and ileum. | Zulkifle et al (2023)^63^ |

Supplementary material F: Luminal signal intensity appearance *in vivo* as reported with direct quotes from the references’ literature

| NMOC media | Intensity effect | | References |
| --- | --- | --- | --- |
| Acai juice | **T1w** | ‘High signal intensity’ on T1 weighted sequences | Arruda-Sanchez et al. (2006)^68^ |
|  |  | ‘Signal enhancement/increase’ and ‘positive characteristic’ | Cordova-Fraga et al (2004)^38^ |
|  | **T2w** | ‘Low signal intensity’ on T2 weighted sequences | Arruda-Sanchez et al. (2006)^68^ |
|  |  | ‘Opacification’ or ‘negative appearance’ | Cordova-Fraga et al (2004)^38^ |
|  |  | Described as ‘hypo’ and ‘lower intensity’. | Pinho et al (2019)^69^ |
|  |  | Described as a ‘negative oral contrast agent’ which could ‘reduce local brightness.’ | Sanchez et al (2009)^70^ |
| Pineapple juice | **T1w** | ‘Bright’ on T1 weighted sequences (positive contrast medium) | Arthurs et al. (2014)^35^ |
|  |  | ‘Bright lumen’ | Asbach et al (2006)^36^ |
|  | **T2w** | ‘Low signal’ on T2 weighted sequences | Arthurs et al. (2014)^35^ |
|  |  | ‘Bright to moderately dark lumen* depending on the concentration of the paramagnetic substance (e.g., manganese concentration in different juices and the TE of the MRI sequence).’ | Asbach et al (2006)^36^ |
|  |  | ‘Effective signal suppression in the GI tract’ reported in the discussion but only pictorial data presented in the results without a description. | Elsayed et al (2015)^41^ |
|  |  | ‘Negative’ reported in the introduction and discussion sections, but signal intensity of regions of interest demonstrated a decreased signal intensity on T2 weighted imaging. | Mohabir et al (2020)^61^ |
|  |  | Pineapple juice described as a ‘negative oral contrast agent’ in the introduction and discussion with outcomes measured in terms of enhancement of the bilary lumen. ‘Regions of interest were used to measure the signal intensity of the duodenal lumen and the gastric fundus lumen relative to the gallbladder or biliary tract, cerebrospinal fluid or renal cyst content.’ | Renzulli et al (2022)^62^ |
|  |  | Described as a ‘negative oral contrast agent’ exhibiting ‘gastroinestinal fluid signal suppression.' | Renzulli et al (2019)^17^ |
|  |  | Described as a ‘negative oral contrast’. | Riordan et al (2004)^13^ |
| Blueberry juice | **T1w** | ‘Bright lumen’ | Asbach et al (2006)^36^ |
|  |  | ‘The signal intensity of blueberry juice at all tested concentrations was nearly equal’ (less than or equal) ‘to subcutaneous fat, indicating positive-contrast enhancement of the stomach contents.’ | Hiraishi et al (1995)^65^ |
|  |  | ‘The signal intensity of blueberry juice in the stomach was almost equal to subcutaneous fat. and acting a positive contrast agent. The signal intensity of the blueberry juice in the stomach was more conspicuous on fat-suppressed echo-planar images because of the scaling effect. Although not obvious on the qualitative evaluation, there was a statistically significant signal increase in all parts of the proximal gastrointestinal tract.’ | Karantanas et al (2000)^66^ |
|  |  | ‘Intermediate signal intensity’ and a ‘positive contrast agent’ in the discussion. | Lam et al (2007)^48^ |
|  | **T2w** | ‘Bright to moderately dark lumen* depending on the concentration of the paramagnetic substance (e.g., manganese concentration in different juices and the echo time (TE) of the MRI sequence).’ | Asbach et al (2006)^36^ |
|  |  | ‘On T2-weighted images, the signal intensity of blueberry juice at manganese concentrations of 3.0 mg/dL is below that of water, which indicated that blueberry juice with concentrations of manganese greater than 3.0 mg/dL acts as a negative contrast agent in humans.' | Hiraishi et al (1995)^65^ |
|  |  | ‘The T2 weighted sequence showed no obvious signal loss of the gastrointestinal contents, but there was a statistically significant difference after blueberry juice administration in the duodenum.’ | Karantanas et al (2000)^66^ |
|  |  | ‘The signal intensity of duodenum was decreased statistically significantly… in post blueberry juice sequences, avoiding overlap with the common bile duct’. It was also described as a ‘negative contrast agent for suppressing the signal of the stomach and duodenum’ in the discussion. | Papanikolaou et al (2000)^67^ |
| Water | **T1w** | ‘Dark lumen’ | Asbach et al (2006)^36^ |
|  |  | ‘Low signal intensities’ and a ‘negative contrast agent in the discussion.’ | Lam et al (2007)^48^ |
|  |  | ‘Hypointense’ or producing a ‘dark lumen’. | Scheibl et al (2005)^52^ |
|  |  | ‘Low in signal intensity relative to both adjacent fat and liver.’ | Wesbey et al (1985)^56^ |
|  |  | ‘Hypointense’/’low signal intensity’. | Zhong et al (2005)^58^ |
|  | **T2w** | ‘Bright lumen’ | Asbach et al (2006)^36^ |
|  |  | ‘Positive’ on TrueFISP | Cronin et al (2009)^40^ |
|  |  | ‘Positive/bright contrast medium’ on ‘modulus blipped echo-planar single pulse technique’ | Evans et al (1993)^59^ |
|  |  | ‘Bright’ on a TrueFISP sequence. | Lauenstein et al (2003)^16^ |
|  |  | ‘High signal intensity’ | Minowa et al (1999)^50^ |
|  |  | ‘Bright.’ | Wright et al (1999)^60^ |
|  |  | ‘High signal intensity.’ | Zhong et al (2005)^58^ |
| Orange juice | **T1w** | ‘Dark lumen’ | Asbach et al (2006)^36^ |
|  | **T2w** | ‘Bright lumen’ | Asbach et al (2006)^36^ |
| Infant feeding formula | **T1w** | ‘High signal intensity’ | Balzarini et al (1992)^73^  Bisset (1989) ^37^ |
|  |  | ‘High intensity signal’ and a ‘positive agent’. ‘Enfamil-Iron-MCT had a slightly stronger signal on T1 than the other formulas and slightly weaker signal on T2 that the other formulas.’ Similac-20… providing intermediate signal intensities on T1 weighted images.’ | Gerscovich et al (1990)^43^ |
|  | **T2w** | ‘High signal intensity’ | Bisset (1989)^37^ |
|  |  | ‘High intensity signal’ and a ‘positive agent’. ‘Enfamil-Iron-MCT had a slightly stronger signal on T1 than the other formulas and slightly weaker signal on T2 that the other formulas.’ | Gerscovich et al (1990)^43^ |
|  | **PDw** | ‘High signal intensity’ | Bisset (1989)^37^ |
| Medlar *(Achras Sapota* L.) | **T1w** | ‘Negative behaviour’ and ‘demonstrated a similar intensity to water’) | Cordova-Fraga et al (2012)^39^ |
|  | **T2w** | ‘Positive behaviour’ and demonstrated a similar intensity to water | Cordova-Fraga et al (2012)^39^ |
| Blackberry (*Rubus* spp.) | **T1w** | ‘Positive’ | Espinosa et al (2006)^42^ |
|  | **T2w** | ‘Negative’ | Espinosa et al (2006)^42^ |
| Black tea | **T2w** | ‘Negative’ | Ghanaati et al (2011)^75^ |
|  |  | ‘Negative’, however only a conference abstract. | Videira et al (2016)^76^ |
| Date syrup | **T2w** | ‘Hypointense’ | Govindarajan et al (2014)^72^ |
| Yerba mate (*Ilex paraguayensis*) | **T1w** | ‘Increase in signal intensity in the gastric lumen.’ ‘Hyperintensity of the gastric lumen’ (T1w). | Nestle et al (2006)^78^ |
|  | **T2w** | ‘Images clearly show the biphasic nature of the yerba mate contrast agent.’ |  |
| Oxygen super saturated water | **T1w** | ‘Hyperintense appearance.’ | Nestle (2004)^51^ |
| Oil emulsion | **T1w** | ‘Approaches isointensity with the bowel wall and adjacent soft tissue’ and ‘…relatively low positive contrast effect on T1SE.’ | Tart et al (1991)^74^ |
|  | **T2w** | 'Positive.’ | Tart et al (1991)^74^ |
| Jasmine tea | **T2w** | ‘Negative’ | Utami et al (2021)^55^ |
| Roselle tea (*Hibicus sabdariffa* Linn.) | **T2w** | ‘Negative’ | Varavithya et al (2005)^77^ |
